# Prediction of tricuspid regurgitation regression after mitral valve transcatheter edge-to-edge repair using three-dimensional transoesophageal echocardiography

**DOI:** 10.1093/ehjimp/qyaf016

**Published:** 2025-01-29

**Authors:** Makoto Takeuchi, Hiroto Utsunomiya, Kiyotaka Tohgi, Ayano Hamada, Yohei Hyodo, Akane Tsuchiya, Atsuo Mogami, Hajime Takemoto, Kanako Izumi, Kosuke Takahari, Yusuke Ueda, Kiho Itakura, Hiroki Ikenaga, Yukiko Nakano

**Affiliations:** Department of Cardiovascular Medicine, Hiroshima University Graduate School of Biomedical and Health Sciences, Hiroshima 734-8551, Japan; Department of Cardiovascular Medicine, Hiroshima University Graduate School of Biomedical and Health Sciences, Hiroshima 734-8551, Japan; Department of Cardiovascular Medicine, Hiroshima University Graduate School of Biomedical and Health Sciences, Hiroshima 734-8551, Japan; Department of Cardiovascular Medicine, Hiroshima University Graduate School of Biomedical and Health Sciences, Hiroshima 734-8551, Japan; Department of Cardiovascular Medicine, Hiroshima University Graduate School of Biomedical and Health Sciences, Hiroshima 734-8551, Japan; Department of Cardiovascular Medicine, Hiroshima University Graduate School of Biomedical and Health Sciences, Hiroshima 734-8551, Japan; Department of Cardiovascular Medicine, Hiroshima University Graduate School of Biomedical and Health Sciences, Hiroshima 734-8551, Japan; Department of Cardiovascular Medicine, Hiroshima University Graduate School of Biomedical and Health Sciences, Hiroshima 734-8551, Japan; Department of Cardiovascular Medicine, Hiroshima University Graduate School of Biomedical and Health Sciences, Hiroshima 734-8551, Japan; Department of Cardiovascular Medicine, Hiroshima University Graduate School of Biomedical and Health Sciences, Hiroshima 734-8551, Japan; Department of Cardiovascular Medicine, Hiroshima University Graduate School of Biomedical and Health Sciences, Hiroshima 734-8551, Japan; Department of Cardiovascular Medicine, Hiroshima University Graduate School of Biomedical and Health Sciences, Hiroshima 734-8551, Japan; Department of Cardiovascular Medicine, Hiroshima University Graduate School of Biomedical and Health Sciences, Hiroshima 734-8551, Japan; Department of Cardiovascular Medicine, Hiroshima University Graduate School of Biomedical and Health Sciences, Hiroshima 734-8551, Japan

**Keywords:** 3D-echocardiography, mitral valve transcatheter edge-to-edge repair, tricuspid valve annulus area, tricuspid valve annulus perimeter, tricuspid regurgitation

## Abstract

**Aims:**

We aimed to identify three-dimensional echocardiographic predictors of tricuspid regurgitation (TR) regression in patients with functional TR of moderate or greater severity undergoing mitral valve transcatheter edge-to-edge repair to optimize patient selection and improve clinical outcomes.

**Methods and results:**

This retrospective study analysed 61 patients (mean age 81.3 ± 7.6 years; 55.7% males) who underwent mitral valve transcatheter edge-to-edge repair. Two-dimensional transthoracic echocardiography was performed pre- and 1-month post-procedurally, while three-dimensional transoesophageal echocardiography was performed pre-procedurally. We collected data on clinical variables, medications, and detailed echocardiographic measurements to evaluate procedural outcomes. Tricuspid regurgitation severity was semiquantitatively assessed and categorized. At the 1-month follow-up, TR severity had regressed in 43% of patients. A lower prevalence of atrial fibrillation, smaller left atrial volume index, and smaller right atrial area were significantly associated with TR regression. Multivariate analysis revealed the tricuspid valve annulus perimeter, area, and area change as significant predictors of post-procedure TR regression; tricuspid valve annulus perimeter was the strongest predictor among the three indicators [area under the receiver operating characteristic curve, 0.84 (95% confidence interval: 0.75–0.94), *P* < 0.001]. Receiver operating characteristic curve analysis indicated that tricuspid valve annulus perimeter cut-off of ≤13.75 cm was the best predictor of post-procedure TR regression. Additionally, tricuspid valve annulus area ≤13.55 cm² and annulus area change ≥17.5% were predictors of post-procedure TR regression.

**Conclusion:**

In patients with relatively severe mitral regurgitation with a non-dilated tricuspid annulus and significant change in tricuspid valve annulus area, mitral valve transcatheter edge-to-edge repair may lead to TR regression.

## Graphical Abstract

**graphical abstract:**
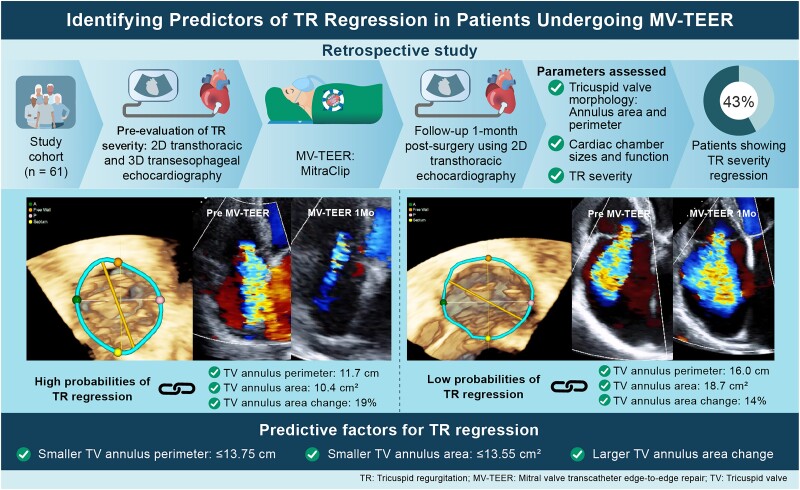
Identifying predictors of TR regression in patients undergoing MV-TEER

## Introduction

Tricuspid regurgitation (TR) is often secondary to left-sided valvular diseases, such as mitral or aortic valve disorders, accounting for 49.5% of all TR cases.^1^ Tricuspid regurgitation prevalence is higher in females and increases with age.^2^ Tricuspid regurgitation is frequently associated with chronic atrial fibrillation (AF) and intracardiac devices in patients with arrhythmia and heart failure (HF).^3^ Moderate or greater TR is reported in 33.6% of adult patients with newly diagnosed AF, with 10.6% classified as isolated TR without significant underlying heart disease.^4^ Thus, TR in valvular disorders of the left heart is complex and multifactorial but often progresses asymptomatically.^5^ Moderate-to-severe TR is associated with increased mortality and hospitalization for worsening HF within 1 year after mitral valve transcatheter edge-to-edge repair (MV-TEER).^6^ Indeed, patients with mitral regurgitation (MR) with severe TR were excluded from the MITRA-FR and COAPT trials.^7,8^ However, recent advancements have made transcatheter tricuspid valve (TV) edge-to-edge repair (TV-TEER) feasible for this patient group, leading to significant improvements in HF symptoms, as measured using the New York Heart Association (NYHA) functional classification.^9,10^ The TRILUMINATE trial demonstrated that TV-TEER reduced TR severity by ≥1 grade in 86% of patients within 30 days post-procedure,^11^ suggesting that predicting TR regression before MV-TEER could benefit those with severe TR by facilitating a tailored and effective treatment approach.

To our knowledge, only one study has reported that factors such as AF; moderate or severe post-procedural residual MR; and tricuspid annular diameter, particularly measurements of ≥34 mm, hinder TR improvement.^12^ Therefore, comprehensive studies are needed to identify three-dimensional (3D) echocardiographic parameters contributing to TR reduction after MV-TEER for optimizing patient selection and improving clinical outcomes. We aimed to explore predictors of TR regression in patients with moderate or significant TR undergoing MV-TEER, focusing on 3D-TV parameters. Our goal is to improve patient outcomes and provide better guidance for clinical decision-making in managing TR secondary to left-sided valvular disease.

## Methods

### Study population

**Figure 1:**
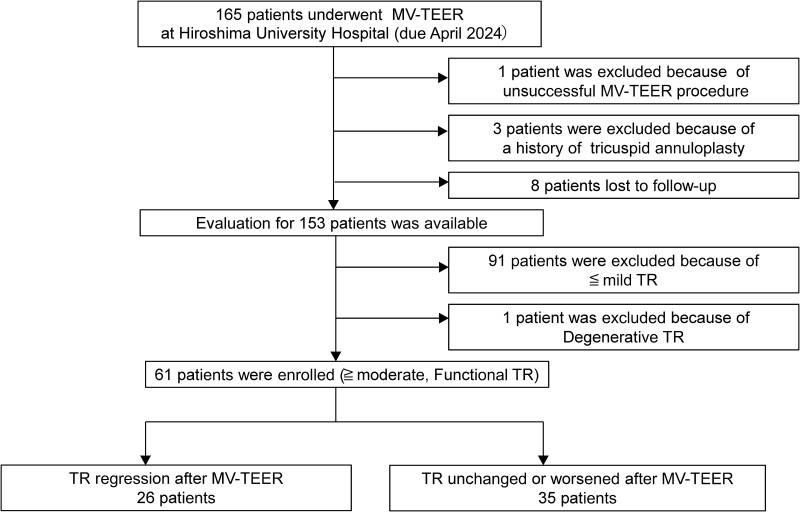
Flowchart illustrating the inclusion/exclusion of study participants.

This retrospective study included 165 consecutive patients who underwent MV-TEER at Hiroshima University Hospital (Hiroshima, Japan) between June 2019 and April 2024. The Ethics Committee of Hiroshima University Hospital waived the requirement for ethics approval and informed consent owing to the study's retrospective nature. The study was performed in accordance with the Declaration of Helsinki and its later amendments. We excluded one patient owing to an unsuccessful edge-to-edge repair procedure, three owing to a history of tricuspid annuloplasty, and eight who were lost to follow-up. All patients underwent comprehensive evaluation using two-dimensional (2D) transthoracic echocardiography (TTE) and 3D-transoesophageal echocardiography (TEE) immediately before the procedure. When possible, 2D-TTE and 3D-TEE were performed on the same day; if not, 3D-TEE was performed within 5 days of 2D-TTE. The initial TR severity was graded following the current guidelines^13^ and subsequently reclassified based on updated criteria using average vena contracta (VC) measurement in biplane views.^14^ We categorized TR into five grades: mild, moderate, severe, massive, and torrential. Of the patients who had undergone MV-TEER operation in our hospital, 91 were excluded because their TR was graded as mild or less and one because of degenerative TR. Sixty-one patients were included in the final cohort. To assess outcomes, these patients underwent follow-up with 2D-TTE at 1 month after MV-TEER. Of these, 26 showed TR regression and 35 showed unchanged TR or progression of TR severity (*Figure 1*).

### Clinical variables

Baseline data were retrospectively collected, including demographic information, medical history, comorbidities, and details of HF medications before and after MV-TEER, such as angiotensin-converting enzyme inhibitors, angiotensin II receptor blockers, angiotensin receptor neprilysin inhibitors, beta-blockers, aldosterone antagonists, and sodium-glucose cotransporter 2 inhibitors, as well as diuretic dosages. The dosages of loop diuretics were converted to furosemide-equivalents and documented at initial 2D-TTE and 1-month post-MV-TEER. The estimated glomerular filtration rate was calculated using the Modification of Diet in Renal Disease formula.^15^ MR aetiology was classified as atrial functional MR (AFMR), ventriculogenic functional MR (VFMR), or degenerative MR. Atrial functional MR is characterized by normal left ventricular (LV) size and preserved function [LV ejection fraction (LVEF) ≥ 50%], significant left atrial (LA) dilation [LA volume index (LAVI) ≥ 40 mL/m²], and mitral annular dilation with a normal leaflet appearance. Conversely, VFMR is characterized by a dilated LV with global or regional dysfunction (LVEF, often <50%), papillary muscle displacement causing leaflet tethering, and mitral annular dilation in response to LA dilation.^16^

### 2D-TTE and 3D-TEE

Two-dimensional-TTE data were acquired with patients at rest using an ultrasound system (EPIQ7; Philips, Andover, MA, USA) equipped with S5-1 transducers. Similarly, 3D-TEE was performed using an EPIQ7 system with a fully sampled matrix array transducer (X8-2t Live 3D-TEE transducer; Philips), allowing for high-volume 3D-imaging. Additional details are provided in Supplementary Methods.^17–20^

### 3D-TV analysis

**Figure 2:**
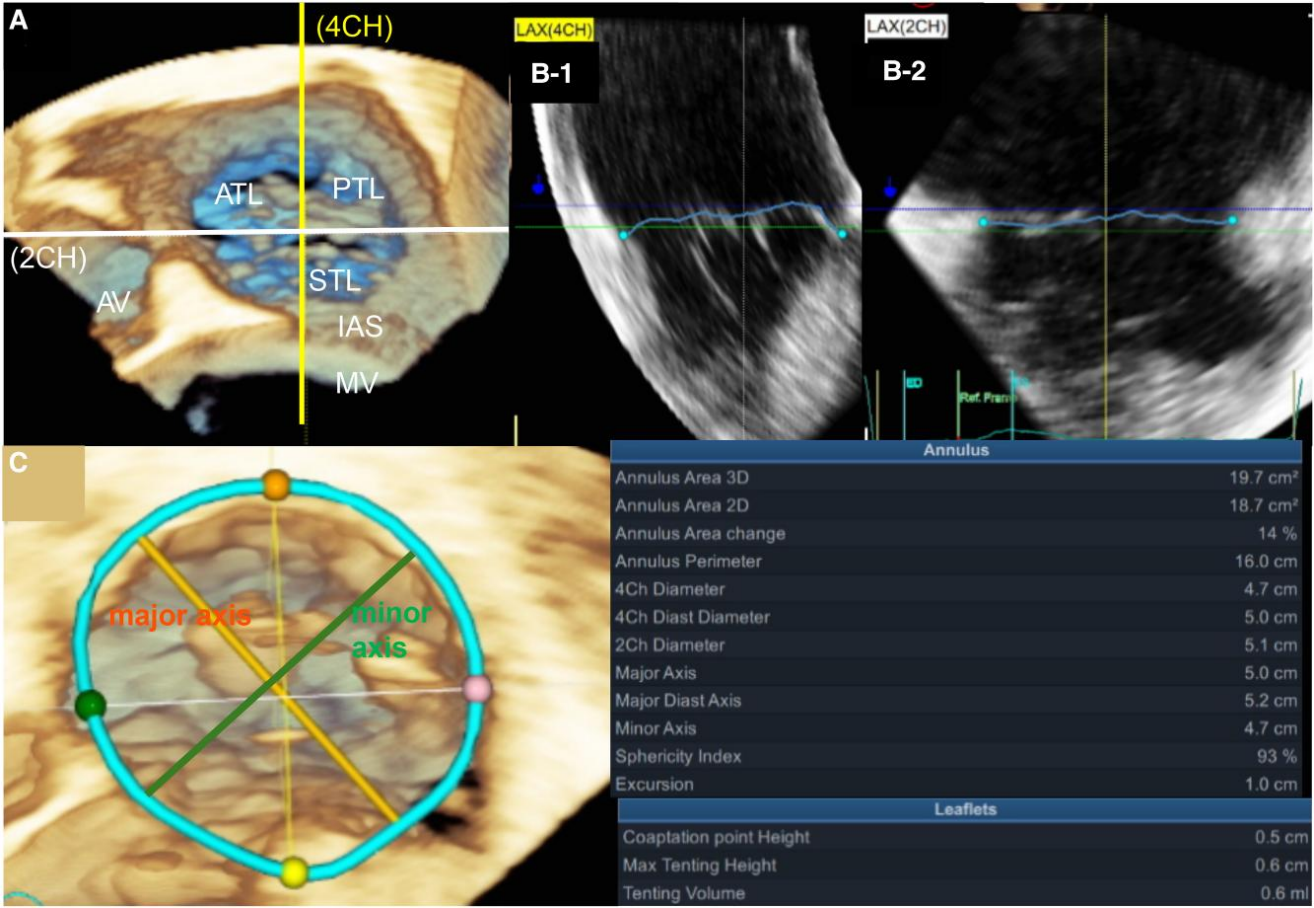
Evaluation of tricuspid valve parameters using 3D-TEE. *(A*) Tricuspid valve and surrounding structures visible on 3D-TEE, (*B*) Determining four-chamber (B-1) and two-chamber views (B-2) based on surrounding structures, and (*C*) Automatically calculated tricuspid valve parameters. ATL, anterior tricuspid leaflet; STL, septal tricuspid leaflet; PTL, posterior tricuspid leaflet; AV, aortic valve; IAS, atrial septum; 4ch, 4-chamber view; 2CH, 2-chamber view.

The digitally stored 3D-zoom dataset was imported into the 4D Auto TVQ application on the Vivid Ultra Edition (GE Healthcare, Chicago, IL, USA; *Figure 2*). Tricuspid valve segmentation was performed using a comprehensive multi-step approach. Additional details are provided in Supplementary Methods.^21–23^ This study used specific terms to describe the various metrics related to the annulus and leaflets. The dimensions, including ‘major axis,’ ‘minor axis,’ ‘4ch diameter,’ ‘2ch diameter,’ and ‘4ch diastolic diameter,’ refer to direct measurements of the annulus. ‘Max tenting height’ and ‘tenting volume’ describe leaflet characteristics. For clarity and consistency, ‘annulus area change’ refers to the ‘TV annulus area change,’ while ‘annulus perimeter’ denotes the ‘TV annulus perimeter.’ Among the available metrics (‘annulus area 2D’ and ‘annulus area 3D’), we selected ‘annulus area 2D,’ which we have termed the ‘TV annulus area’ owing to its broader clinical application. This measurement represents a 3D-TV structure projected onto a 2D plane. The TV annulus area change (%) was calculated as [(Maximum area–Minimum area)/Maximum area] × 100.

### MV-TEER procedure and follow-up

MV-TEER procedures were performed under general anesthesia with fluoroscopic and TEE guidance. MitraClip devices (Abbott Cardiovascular, Chicago, IL, USA) were used, and the procedures were performed by a single operator in all cases. The procedure began with a transseptal puncture. The MitraClip device was advanced into the left atrium and then into the left ventricle to grasp the MV leaflets, thereby reducing regurgitation.^24^ Transthoracic echocardiography examinations were conducted before MV-TEER treatment and at the 1-month follow-up.

### Statistical analysis

We conducted a power analysis for the Mann–Whitney *U t*est and determined the required sample size for our study as 20 and 24 when using the TV annulus perimeter and area, respectively. Categorical variables are expressed as numbers and percentages. Continuous data are presented as mean ± standard deviation for normally distributed variables and as median with interquartile range for non-normally distributed variables. Pearson's χ^2^ test was performed to compare two categorical variables, and the *t*-test and Mann–Whitney *U* test were performed to compare continuous variables with normally and non-normally distributed data, respectively. To investigate changes in HF medication and diuretic dosage before and after surgery, McNemar's test was used for categorical variables, while Wilcoxon signed-rank test was used for continuous variables.

The optimal cut-off values for the TV annulus perimeter, area change, and area were determined using receiver operating characteristic (ROC) analysis, which allowed identification of cut-off points at which sensitivity and specificity were maximized to categorize variables accurately and to enhance the predictive power of the models used in this study. Multivariate logistic regression analysis was performed to control for potential confounding variables. Variables were included in this analysis based on their significance (*P* < 0.2) in univariate analysis or their biological plausibility. To ensure comparability across variables with different scales, each factor included in logistic regression analysis was standardized using *Z*-scores, to allow for the interpretation of the relative importance of each variable within the model. A *P* value <0.05 was considered statistically significant. All statistical analyses were performed using SPSS version 26 for Windows (IBM Corp., Armonk, NY, USA).

## Results

### Patient characteristics

*Table 1* summarizes the baseline patient characteristics of the final cohort (*n* = 61). The mean patient age was 81.3 ± 7.6 years [34 males (55.7%)]. The patients had chronic HF symptoms of NYHA class III (median). The causes of MR were classified as 16 (26.2%) patients with primary and 45 (73.8%) with secondary MR.^13^ Of those with secondary causes, 26 (57.8%) were atrial functional and 19 (42.2%) were ventriculogenic in nature. The mean number of clips placed using MV-TEER was 1.05 ± 0.22. Atrial fibrillation was detected in 49 patients (80.3%). Among the 61 patients, the MitraClip XTW system was used in 27 (44.3%), NT system in 23 (37.7%), XT system in 7 (11.5%), and NTW system in 4 (6.6%). Postoperative moderate or greater MR was observed in 9.8% (6/61) of patients. Additionally, no significant differences in post-operative MR severity were found in the TR regression or control group (11.4% vs. 7.6%, *P* = 0.38). The baseline initiation rates of heart failure medications showed no differences between the two groups. When comparing the initiation rates of heart failure medications before and after MV-TEER, no significant differences were observed in any medication between the TR regression and control groups (*P* = 0.25–1.00). Additionally, the changes in loop diuretic dosages were evaluated, but no significant differences were found in the TR regression (*P* = 0.28) or control group (*P* = 0.93) before and after the procedure. Similarly, the changes in tolvaptan dosages were analyzed, and no significant differences were identified in either the TR regression (*P* = 0.40) or control group (*P* = 0.63). There was no significant difference in 2D-TTE parameters except for LAVI and right atrial area; the preoperative stroke volume was 46.0 ± 16.0 mL in the TR regression group and 52.3 ± 14.7 mL in the control group (*P* = 0.12). Post-operatively, it was 56.0 ± 19.1 and 52.6 ± 16.5 mL, respectively (*P* = 0.46), with no significant differences. The preoperative tricuspid annular plane systolic excursion (TAPSE) to systolic pulmonary arterial pressure (sPAP) ratio was 0.34 ± 0.11 vs. 0.37 ± 0.17 mm/mm Hg (*P* = 0.32), and 0.47 ± 0.17 vs. 0.40 ± 0.14 mm/mm Hg (*P* = 0.25) post-operatively, with no significant differences.

**Table 1:** Clinical characteristics of the study population and according to TR regression

	Total (*n* = 61)	TR regression (*n* = 26)	TR unchanging or progression (*n* = 35)	*P* value
Age (years)	81.3 ± 7.6	81.7 ± 5.7	81.1 ± 8.8	0.75
Sex (male)	34 (55.7%)	12 (46.2%)	22 (62.9%)	0.19
Atrial fibrillation	49 (80.3%)	17 (65.4%)	32 (91.4%)	0.01
Hypertension	26 (42.6%)	13 (50%)	13 (37.1%)	0.32
Diabetes mellitus	13 (32.8%)	7 (26.9%)	6 (17.1%)	0.85
Coronary artery disease	14 (23.0%)	8 (30.8%)	6 (17.1%)	0.21
Chronic obstructive pulmonary disease	4 (6.6%)	0 (0%)	4 (11.4%)	0.08
Previous myocardial infraction	7 (11.5%)	5 (19.2%)	2 (5.7%)	0.10
Previous coronary artery bypass grafting	5 (8.2%)	2 (7.7%)	3 (8.6%)	0.90
Permanent pacemaker	12 (19.7%)	4 (15.4%)	8 (23.5%)	0.43
Haemodialysis	6 (9.8%)	2 (7.7%)	4 (11.4%)	0.63
New York Heart Association Class	3 (3)	3 (3)	3 (3)	0.48
MR aetiology				0.006
Degenerative	16 (26.2%)	9 (34.6%)	7 (20.0%)	
Atrial functional	26 (42.6%)	5 (19.2%)	21 (60.0%)	
Ventriculogenic functional	19 (31.1%)	12 (46.2%)	7 (20.0%)	
Preoperative medications				
Angiotensin-converting enzyme inhibitor or angiotensin II receptor blocker or angiotensin receptor neprilysin inhibitor	38 (62.3%)	16 (61.5%)	22 (62.9%)	0.92
Beta-blocker	50 (82.0%)	20 (76.9%)	30 (85.7%)	0.38
Aldosterone antagonist	36 (59.0%)	17 (65.4%)	19 (54.3%)	0.38
Sodium-glucose cotransporter 2 inhibitor	28 (45.9%)	13 (50.0%)	15 (42.9%)	0.58
Pre dosage of loop diuretics (mg/day)	29.1 ± 30.8	24.2 ± 22.1	32.7 ± 35.8	0.29
Pre dosage of tolvaptan (mg/day)	6.6 ± 5.5	6.9 ± 5.5	6.4 ± 5.6	0.73
Postoperative medications				
ACEi or ARB or ARNI	41 (67.2%)	17 (65.4%)	24 (68.6%)	0.79
Beta-blocker	52 (85.2%)	22 (84.6%)	30 (85.7%)	0.91
Aldosterone antagonist	38 (62.3%)	18 (69.1%)	20 (57.1%)	0.34
Sodium-glucose cotransporter 2 inhibitor	31 (50.8%)	16 (61.5%)	15 (42.9%)	0.24
Post-dosage of loop diuretics (mg/day)	27.3 ± 28.2	21.5 ± 18.3	31.6 ± 33.3	0.21
Post-dosage of tolvaptan (mg/day)	6.7 ± 5.6	6.2 ± 5.5	7.1 ± 5.7	0.55
Laboratory data				
Haemoglobin (g/dL)	11.4 ± 1.8	11.8 ± 2.0	11.2 ± 1.7	0.15
eGFR (mL/min/1.73 m^2^)	32.9 ± 15.9	34.0 ± 16.3	32.1 ± 15.8	0.66
Log NTproBNP (pg/mL)	3.6 ± 0.6	3.5 ± 0.6	3.6 ± 0.5	0.41
MR grade				0.12
Moderate-to-severe	12 (19.7%)	6 (23.1%)	6 (17.2%)	
Severe	42 (68.9%)	20 (76.9%)	22 (62.9%)	
Follow-up MR grade (≧moderate)	6 (9.8%)	4 (11.4%)	2 (7.6%)	0.38
TR grade				0.93
Moderate	31 (50.8%)	13 (50%)	18 (51.4%)	
Severe	27 (44.3%)	12 (46.2%)	15 (42.9%)	
Massive	3 (4.9%)	1 (3.8%)	2 (5.7%)	
Echo parameters				
Left ventricular end-diastolic diameter (cm)	5.4 (4.9–5.9)	5.5 (5.2–5.9)	5.2 (4.9–5.9)	0.38
Left ventricular end-systolic diameter (cm)	3.8 (3.3–4.8)	3.8 (3.4–4.9)	3.8 (3.3–4.8)	0.61
Left ventricular ejection fraction (%)	51.2 ± 14.5	51.5 ± 16.5	51.1 ± 13.1	0.92
Left atrial volume index (mL/m^2^)	93.3 ± 56.2	73.5 ± 23.8	108.0 ± 68.1	0.02
Stroke volume (mL)	49.6 ± 15.5	46.0 ± 16.0	52.3 ± 14.7	0.12
Right atrial area (cm^2^)	27.3 (21.9–32.5)	23.7 (18.9–28.0)	31.0 (22.5–38.4)	0.002
Right ventricular end-diastolic area (cm^2^)	19.9 (16.4–25.1)	20.0 (15.3–24.8)	19.8 (17.9–24.7)	0.25
Right ventricular end-systolic area (cm^2^)	12.6 (9.9–17.7)	12.8 (9.2–17.8)	12.6 (10.4–17.5)	0.60
Right ventricular fractional area change (%)	36.0 (29.3–42.8)	36.0 (28.0–43.0)	35.5 (30.0–41.8)	0.91
Systolic pulmonary artery pressure (mm Hg)	52.7 ± 16.7	57.4 ± 17.5	48.7 ± 15.2	0.07
TAPSE (mm)	17.2 ± 5.0	17.6 ± 4.4	16.8 ± 5.4	0.52
TAPSE to sPAP ratio (mm/mm Hg)	0.36 ± 0.15	0.34 ± 0.11	0.37 ± 0.17	0.32
TVQ parameters				
TV annulus area (cm^2^)	13.2 (10.9–15.7)	11.1 (9.6–13.3)	14.0 (12.3–18.2)	<0.001
TV annulus area change (%)	15.0 (11.0–19.0)	18.0 (15.0–21.0)	12.0 (10.0–16.0)	0.001
TV annulus perimeter (cm)	13.4 (12.3–14.9)	12.3 (11.4–13.4)	14.2 (13.2–15.6)	<0.001
Major axis (cm)	4.4 (4.0–4.8)	4.1 (3.7–4.4)	4.5 (4.3–5.0)	<0.001
Minor axis (cm)	3.7 (3.4–4.3)	3.5 (3.0–3.7)	4.0 (3.5–4.6)	<0.001
4ch diameter (cm)	4.1 (3.8–4.7)	3.7 (3.4–4.2)	4.4 (4.0–4.8)	<0.001
4ch diastolic diameter (cm)	4.2 (4.1–4.8)	4.2 (3.9–4.3)	4.6 (4.1–4.9)	0.01
2ch diameter (cm)	3.8 (3.6–4.5)	3.6 (3.3–3.9)	4.1 (3.8–4.7)	<0.001
Excursion (cm)	0.78 ± 0.35	0.77 ± 0.38	0.79 ± 0.33	0.78
Max tethering height (cm)	0.64 ± 0.22	0.62 ± 0.24	0.66 ± 0.19	0.46
Tenting volume (mL)	1.2 ± 1.1	1.2 ± 1.2	1.2 ± 1.1	0.80

Categorical variables are expressed as numbers (percentages). Continuous data are presented as mean ± standard deviation for normally distributed variables and median with interquartile range for non-normally distributed variables. ACEi, angiotensin-converting enzyme inhibitor; ARB, angiotensin II receptor blocker; ARNI, angiotensin receptor-neprilysin inhibitor; eGFR, estimated glomerular filtration rate; MR, mitral regurgitation; sPAP, systolic pulmonary artery pressure; TAPSE, tricuspid annular plane systolic excursion; TR, tricuspid regurgitation; TV, tricuspid valve.

### Changes in TR after MV-TEER

**Figure 3:**
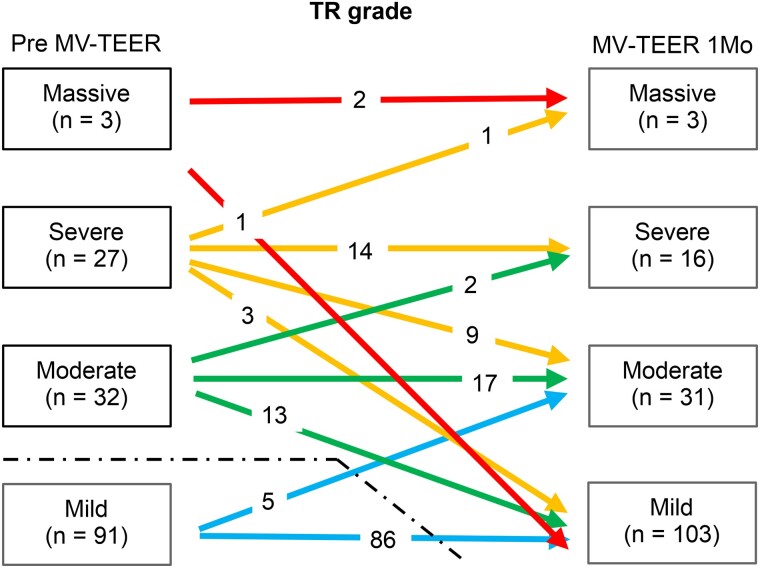
Change in TR severity before and 1 month after MV-TEER. Patients with mild or trivial TR are excluded. Additionally, one patient with preoperative TR is excluded from this study owing to degenerative TR, which remained moderate and unchanged postoperatively. Mo, Month.

The post-procedural changes in TR severity following MV-TEER in the cohort are shown in *Figure 3*. Initially, TR severity was categorized as mild in 91 patients (59%), moderate in 32 (21%), severe in 27 (18%), and massive in 3 (2%), but none had torrential TR. Upon follow-up echocardiographic evaluation, a significant shift in TR distribution post-MV-TEER was observed: 103 patients (67%) exhibited mild TR; 31 (20%), moderate; 16 (10%), severe; and 3 (2%), massive. At the 1-month follow-up, the degree of TR regressed by ≥1 grade in 26 patients (17%) remained unchanged in 119 patients (78%) and progressed by ≥1 grade in 8 patients (5%).

For a more focused analysis, we limited the cohort to patients with moderate or high TR at baseline, resulting in a final cohort of 61 patients (*Table 1*). At the 1-month follow-up, TR severity regressed by ≥1 grade in 26 patients (43%), whereas it remained unchanged or progressed in 35 patients (57%). Notably, baseline TR severity did not differ significantly (*P* = 0.93; *Table 1*).

### Factors associated with TR regression

The prevalence of AF (65.4% vs. 91.4%; *P* = 0.01) and AFMR as the aetiology of MR (19.2% vs. 60.0%; *P* = 0.006) were lower in patients with TR regression than in those without (*Table 1*). The presence of moderate or severe residual MR was similar between the groups (*P* = 0.38). Regarding echocardiographic parameters, the LAVI (73.5 ± 23.8 mL/m^2^ vs. 108.0 ± 68.1 mL/m^2^; *P* = 0.02) and right atrial area were smaller [23.7 cm^2^ (18.9–28.0 cm^2^) vs. 31.0 cm^2^ (22.5–38.4 cm^2^); *P* = 0.002] in patients with TR regression than in those without.

In TVQ analysis, patients with TR regression had a smaller TV annulus perimeter [12.3 cm (11.4–13.4 cm) vs. 14.2 cm (13.2–15.6 cm); *P* < 0.001], higher TV annulus area change [18% (15.0–21.0%) vs. 12% (10.0–16.0%); *P* = 0.001], and smaller TV annulus area [11.1 cm^2^ (9.6–13.3 cm^2^) vs. 14.0 cm^2^ (12.3–18.2 cm^2^); *P* < 0.001] than those without TR regression. Additionally, the diameters of various types of TVs were measured, and the measurement showing the strongest correlation was not the 4ch diastolic diameter (*P* = 0.01), but rather the major axis, which was smaller in patients with TR regression [4.1 cm (3.7–4.4 cm) vs. 4.5 cm (4.3–5.0 cm); *P* < 0.001] than that in patients without, being a predictor of the same magnitude as the TV annulus area. Therefore, the major axis was not included in logistic regression analysis.

In this study, we primarily focused on tricuspid valve morphology; therefore, further analysis regarding the history of AF or aetiology of MR was not conducted. Parameters with significant difference (*P* < 0.2) were tested for their ability to predict improvement in TR using logistic regression analysis. Three multivariate analyses were conducted to evaluate the influence of morphological TV abnormalities and their locations on TR regression (*Table 2*). In the multivariate model, four echocardiographic parameters were included to determine if each factor could independently predict TR regression or if there was an overlap between factors. Standardized *Z*-scores of the TV annulus perimeter, area change, and area; 4ch diastolic diameter; and LAVI were included in the model. Based on the first model, the TV annulus perimeter [odds ratio (OR) = 0.11, 95% confidence interval (CI): 0.03–0.46, *P* = 0.002] and change (OR = 2.89, 95% CI: 1.06–7.92, *P* = 0.04) were significant predictors of TR regression (multivariate model 1). In the second model, the TV annulus area change (OR = 2.70, 95% CI: 1.05–6.94, *P* = 0.04) and area (OR = 0.16, 95% CI: 0.04–0.64, *P* < 0.001) significantly predicted TR regression (multivariate model 2). The third model showed that the TV annulus area change (OR = 3.34, 95% CI: 1.36–8.18, *P* = 0.01) and 4ch diastolic diameter (OR = 0.30, 95% CI: 0.10–0.87, *P* = 0.03) were significant predictors of TR regression. Thus, the TV annulus area change was consistently identified as a significant factor (*P* = 0.01 or 0.04). Based on OR values, the TV annulus perimeter and TV annulus area were superior indicators to the 4ch diastolic diameter, with the TV annulus perimeter being the strongest predictor (*Table 2*).

**Table 2:** Logistic regression model for tricuspid valve regurgitation regression

Variable	Univariate^a^		Multivariate model 1^b^			Multivariate model 2^b^			Multivariate model 3^b^		
	OR (95% CI)	*P* value	OR (95% CI)	*P* value	VIF	OR (95% CI)	*P* value	VIF	OR (95% CI)	*P* value	VIF
Right atrial area (*Z* score^c^)	0.29 (0.12–0.72)	0.01									
TV annulus perimeter^d^ (Z score^c^)	0.10 (0.03–0.34)	<0.001	0.11 (0.03–0.46)	0.002	2.01						
TV annulus area change (*Z* score^c^)	3.49 (1.52–7.97)	0.003	2.89 (1.06–7.92)	0.04	1.38	2.70 (1.05–6.94)	0.04	1.38	3.34 (1.36–8.18)	0.01	1.15
TV annulus area^d^ (*Z* score^c^)	0.13 (0.04–0.40)	<0.001				0.16 (0.04–0.64)	0.01	2.05			
4ch diast diameter^d^ (*Z* score^c^)	0.30 (0.12–0.73)	0.01							0.30 (0.10–0.87)	0.03	1.55
Left atrial volume index (*Z* score^c^)	0.42 (0.19–0.91)	0.03	0.97 (0.45–2.10)	0.94	1.25	0.91 (0.41–2.00)	0.81	1.25	0.60 (0.27–1.36)	0.22	1.09

^a^Trivariate logistic regression analysis adjusted for age and sex for all variables.

^b^Multivariate logistic regression adjusted for age and sex as independent determinants.

^c^Right atrial area, TV annulus perimeter, TV annulus area change, TV annulus area, 4ch diastolic diameter, and left atrial volume index were standardized using Z-scores before inclusion. Standardization was performed for comparability across variables with different scales.

^d^Due to strong multicollinearity, these variables cannot be included simultaneously in the multivariate model.

CI, confidence interval; OR, odds ratio; TV, tricuspid valve; VIF, variance inflation factor.

Finally, as previously reported,^3^ the TV annulus perimeter and TV annulus area were also measured using the QLAB mitral valve navigator software (Philips) on the same images and at the same phase. Robust correlations were observed between the measurements obtained from the two software programs for the TV annulus perimeter (*r* = 0.978, *P* < 0.001) and TV annulus area (*r* = 0.996, *P* < 0.001; see Supplementary data online, *Figure S1*).

### Prediction of TR regression

**Figure 4:**
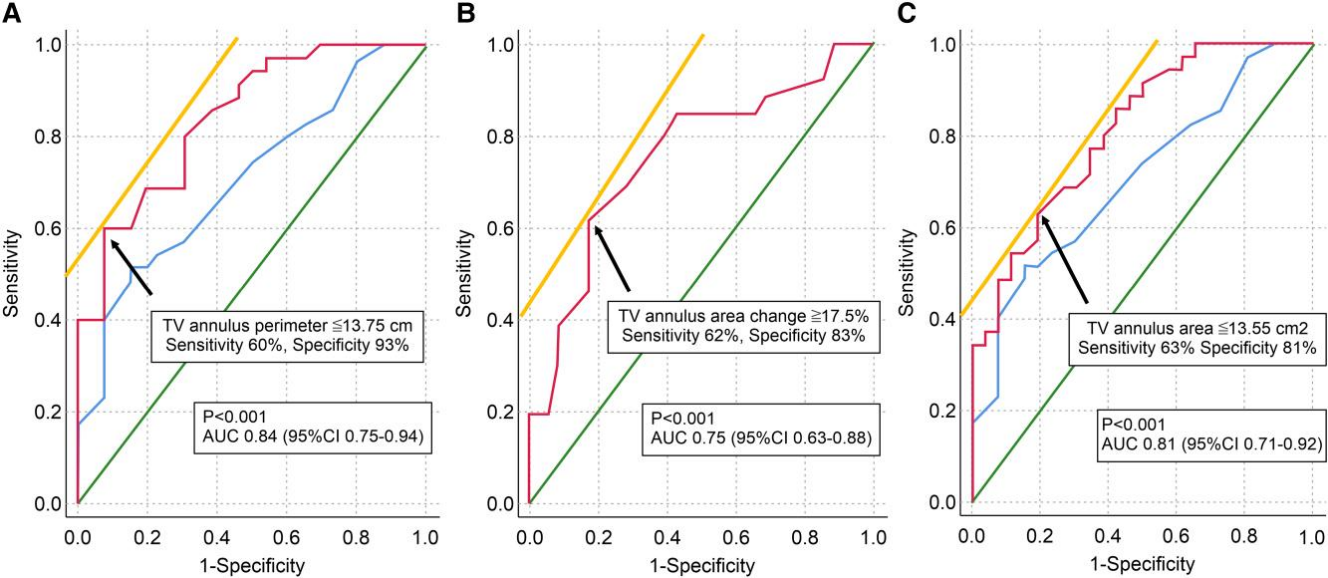
ROC curve analysis showing the cut-off level of (*A*) the TV annulus perimeter (*B*) TV annulus area change, and (*C*) TV annulus area.

Receiver operating characteristic curve analysis was performed using baseline data of patients to predict TR regression using the TV annulus perimeter, area change, and area cut-off values. The area under the ROC curve (AUC) of the TV annulus perimeter was 0.84 (95% CI: 0.75–0.94), *P* < 0.001 (*Figure 4A*), which was superior to that of the 4ch diastolic diameter [0.71 (95% CI: 0.58–0.84), *P* = 0.002] for predicting TR regression. The optimal cut-off value for predicting TR regression was a TV annulus perimeter of ≤13.75 cm (sensitivity 60%; specificity 93%). Additionally, separate analyses were conducted for males and females. Among males (*n* = 34), 12 experienced TR regression, while 22 did not; among females (*n* = 27), 14 had TR regression and 13 did not. The AUC of the TV annulus perimeter was 0.82 (95% CI: 0.68–0.96), *P* = 0.003, with a cut-off value of ≤15.1 cm (sensitivity 59%; specificity 100%) in males and 0.92 (95% CI: 0.82–1.00), *P* < 0.001, with a cut-off value of ≤12.1 cm (sensitivity 100%; specificity 71%) in females. The AUC of the TV annulus area change was 0.75 (95% CI: 0.63–0.88), *P* < 0.001 (*Figure 4B*), with an optimal cut-off value of ≥17.5% (sensitivity 62%; specificity 83%) for predicting TR regression. The AUC of the TV annulus area was 0.81 (95% CI: 0.71–0.92, *P* < 0.001; *Figure 4C*), which was also a significantly superior indicator for predicting TR regression to the 4ch diastolic diameter, based on AUC values. The optimal cut-off value for predicting TR regression was a TV annulus area ≤13.55 cm² (sensitivity 63%; specificity 81%). The AUC of the TV annulus area was 0.78 [(95% CI: 0.62–0.93), *P* = 0.01].

## Discussion

In this study, we aimed to determine the necessity of intervention for TR after MV-TEER; therefore, we utilized the 5-grade classification of TR.^14^ In our study, 40% of patients had moderate or greater functional TR. Of these, 43% (26/61) showed improvement, whereas 57% (35/61) experienced either worsening [5% (3/61)] or unchanged [52% (32/61)] TR after MV-TEER. A smaller TV annulus perimeter, larger TV annulus area change, and smaller TV annulus area were associated with TR regression. The optimal cut-off values for predicting TR regression were a TV annulus perimeter ≤13.75 cm, area change ≥17.5%, and area ≤13.55 cm² (*Graphical Abstract*).

### TR regression after MV-TEER

Patients in whom TR decreased after MV-TEER had lower mortality and HF hospitalization rates at 1 and 2 years than those without TR decrease, with an overall improvement in prognosis.^25^ Additionally, patients in whom TR decreased after MV-TEER for functional MR demonstrated significantly better long-term survival rates at the 1-year follow-up than those without TR decrease.^26^ Therefore, focusing on the changes in TR after MV-TEER is relevant.

The reported changes in TR after MV-TEER vary. According to Kavsur *et al*.^12^ the distribution of preoperative TR severity among cases was 41% mild, 39% moderate, and 19% severe or greater. After 3 months post-operatively, the distribution changed to 49% mild, 35% moderate, and 15% severe (*P* = 0.006).^12^ In our cohort, TR severity distribution at baseline was 59% mild, 21% moderate, and 20% severe or greater, showing somewhat milder cases than in the previous report. However, among patients with moderate or severe TR, the target population of this study, 41% showed TR regression, which was similar to the 43% reported by Kavsur *et al*.^12^ Additionally, they reported significant changes in TR severity within the initial 3 months postoperatively (*P* = 0.006), with only minor changes observed between 3 and 12 months (*P* = 0.813). To consider the potential for TV-TEER, we reassessed the severity of TR at 1 month after MV-TEER, following a similar approach to that in their study.

Toyama *et al*.^27^ reported that TR was improved by ≥1 grade at 1 year after MV-TEER in 23% of patients; moreover, 62% had unchanged and 16% had worsened TR. However, they included trivial TR in their severity assessment, whereas we focused on analyzing moderate or severe TR. When using the same criteria, we noted severe TR in 20% of the patients, moderate in 21%, mild in 45%, and trivial in 14%. The proportion of patients who exhibited TR regression by 1 month after procedure was 24%, which aligned with their reported proportion.

Increased LA pressure due to MR induces pulmonary hypertension, which, in turn, causes right ventricular (RV) dilation and dysfunction.^28^ Subsequently, RV dilatation results in expansion of the tricuspid annulus, leading to TR. This creates a vicious cycle that exacerbates RV dilation and dysfunction. Decreased sPAP may reduce TR, as severe TR resolved in 19 of 27 patients (70%) after pulmonary thromboendarterectomy.^29^ This indicates that severe functional TR can improve after a significant reduction in sPAP without tricuspid annuloplasty.

Additionally, Toyama *et al*.^27^ and Frangieh *et al*.^30^ reported that sPAP reduction was an independent predictor of TR changes after MV-TEER. These findings indirectly supported our observation that TR regression is uncommon in patients with dilated TVs. Since this study aimed to predict TR regression based on pre-MV-TEER parameters, we did not include sPAP reduction in the analysis. However, we found that ΔsPAP (preoperative sPAP−postoperative PAP) was significantly reduced in patients with TR regression when compared with those without TR regression (19.1 ± 14.8 vs. 4.8 ± 15.6 mmHg, *P* = 0.01), suggesting that the reduction in TR may be attributable to an improvement in sPAP. Additionally, the post-operative TAPSE to sPAP ratio improved significantly compared with the pre-operative ratio in the TR regression group (0.14 ± 0.13 vs. 0.05 ± 0.14 mm/mm Hg, *P* = 0.03).

### TV structural determinants of TR regression

We found that a smaller TV annulus perimeter, larger TV annulus area change, and smaller TV annulus area were associated with TR regression. This result supports previous findings indicating that TR regression is less likely to occur when the tricuspid annular diameter is ≥34 mm.^12^ Our measurements for the 4ch diastolic diameter exceeded the reported cut-off values. However, this discrepancy may be attributed to errors in 2D- and 3D-echocardiography measurements. Changing the probe from the apical four-chamber view to the RV-focused view can result in different measurements of the tricuspid annulus.^31^ Greater accuracy of the 4ch diastolic diameter is required, and it may be an inferior predictor of TR regression to the major axis and TV annulus perimeter and area.

The optimal cut-off value for predicting TR regression was a TV annulus perimeter ≤13.75 cm, suggesting that patients with an enlarged atrial secondary tricuspid annulus have a lower risk of TR reduction after MV-TEER that that of those without. Muraru *et al*.^32^ reported that a TA annulus perimeter >13.7 cm in males and >12.5 cm in females suggested atrial secondary TR. Among the cases that met the criteria for atrial secondary TR, 23.1% constituted the TR regression group, whereas 74.3% constituted the control group (*P* < 0.001). In our study, the sex-specific indicators were TV annulus perimeter ≤15.1 cm and ≤12.1 cm in males and females, respectively.

The optimal cut-off value for predicting TR regression was a TV annulus area ≤13.55 cm². Similarly, Muraru *et al*.^32^ reported that a TV annulus area >13.8 cm² for males and >11.7 cm² for females suggested atrial functional TR (AFTR). Additionally, the TV annulus area in patients with secondary AFTR was reported to be ≥14.36 cm² (12.58–19.50 cm²).^31^

Although knowledge regarding the TV annulus area change is limited, one study reported it to be significantly lower in patients with functional TR [8.2% (6.2–10.1%)] than in the standard control group [20.3% (18.2–21.6%)]. It was 11.5% (8.6–14.3%) in the atrial secondary TR group and 8.2% (6.2–10.1%) in the ventricular secondary TR group.^3^ The optimal cut-off value for predicting TR regression was a TV annulus area change ≥17.5%. Therefore, patients with relatively normal tricuspid annulus contractions are more likely to experience TR regression. Thus, we recommend using thresholds of annulus enlargement that are comparable to or slightly more severe than the cut-off values for atrial secondary TR when predicting post-MV-TEER TR regression.

## Clinical implications

Mortality rate increases as the severity of TR increases.^33^ Specifically, the 1-year survival rate was 91.7% in the absence of TR but decreased to 90.3% when TR was mild, 78.9% when it was moderate, and 63.9% when TR was severe. Therefore, if TR persists after MV-TEER, intervention for TR is essential. Identification of the predictors of TR regression may help in making decisions regarding MV-TEER and TV-TEER in the short term. We measured the TV annulus perimeter and area preoperatively using 3D echocardiography to predict whether TR regression would occur. Considering our findings, performing 3D imaging of the tricuspid valve in patients with severe MR and concomitant moderate or greater TR may provide significant benefits. However, these measurements may also serve as benchmark criteria for determining whether TR can be classified as AFTR.

## Limitations

First, this was a retrospective study; a prospective multicenter study is required to provide more robust conclusions. In addition, we included only 61 patients who underwent MV-TEER with moderate or greater TR. Despite the small sample, we believe the evidence is reasonably robust. Second, we utilized a Philips machine to acquire echocardiographic images, while the analysis of the tricuspid valve was performed using GE's proprietary 4D AutoTVQ software. We used 4D AutoTVQ as it enables semi-automated and entire cardiac cycle analysis of tricuspid valve data. Although the compatibility of the two software programs has not been fully established, the measurement errors for the tricuspid annulus parameters obtained from both programs were minimal. Third, the TR grade was semiquantitatively evaluated. A complete quantitative evaluation using the 3D-VC area or the proximal isovelocity surface area method could enhance the accuracy of this study. However, since we did not perform 3D-TTE or 3D-TEE during the study period, we could not attempt such measurements. Finally, follow-up was conducted 1 month after MV-TEER, representing a short-term, single-point assessment. However, research indicates that TR regression mostly occurs within 3 months after MV-TEER,^12^ suggesting that a short-term evaluation may be accurate. Additionally, reassessment of TR after longer follow-up periods, such as 6 months or more, would strengthen these results, allowing the selection of this short interval to observe the potential simultaneous timing of TV-TEER. Further investigations are required to determine the optimal timing for assessing TR regression.

## Conclusions

In patients with severe MR who have a non-dilated tricuspid annulus and a significant change in TV annulus area, MV-TEER is expected to result in tricuspid TR regression.

## Supplementary data

Supplementary data are available at *European Heart Journal - Imaging Methods and Practice* online.

## Supplementary Material

qyaf016_Supplementary_Data

## Data Availability

The datasets used and/or analyzed during the current study are available from the corresponding author on reasonable request.

## Lead author biography

Dr Makoto Takeuchi graduated from Hiroshima University, Japan. He currently works as a clinical staff in the Department of Cardiovascular Medicine at Hiroshima University Hospital, Hiroshima, Japan. Additionally, he specializes in transthoracic and transoesophageal echocardiography, especially in 3-dimensional imaging.
