# Supplementary material for: Prediction of tricuspid regurgitation regression after mitral valve transcatheter edge-to-edge repair using three-dimensional transoesophageal echocardiography
Source: Eur Heart J Imaging Methods Pract. 2025 Jan 29;3(1):qyaf016. doi: 10.1093/ehjimp/qyaf016 (PMC11811635; doi:10.1093/ehjimp/qyaf016)
Supplement: qyaf016_Supplementary_Data [file qyaf016_supplementary_data.docx]

**SUPPLEMENTARY DATA**

**Supplementary Methods**

**Two-dimensional (2D)-transthoracic echocardiography (TTE)**

The 2D-TTE data were acquired with patients at rest using the latest commercially available ultrasound system (EPIQ7, Philips, Andover, MA, USA) equipped with S5-1 transducers. This cutting-edge technology ensured the accuracy of our measurements of left ventricular (LV) end-diastolic diameter and LV end-systolic diameter in the parasternal long-axis view. Left ventricular ejection fraction was calculated using the biplane Simpson method.^17^ Stroke volume was calculated by measuring the left ventricular outflow tract diameter and velocity.^18^ The right ventricular (RV), right atrial dimensions, and tricuspid annular plane systolic excursion were assessed following the current guidelines for echocardiographic evaluation of the right heart.^19^ Systolic pulmonary artery pressure was derived from the gradient calculated using the maximum velocity of tricuspid regurgitation (TR), with the addition of the estimated RA pressure obtained from the diameter and inspiratory collapse of the inferior vena cava.^19^ TR jet was assessed using color Doppler in the RV inflow and apical four-chamber views. TR severity was meticulously evaluated by two cardiologists and one sonographer using Doppler echocardiography, employing a combination of qualitative and semiquantitative parameters for an integrated approach. This included additional proximal isovelocity surface area and vena contracta (VC) width measurements.^20^ VC width was measured in the apical four-chamber and parasternal RV inflow views, with the average VC width calculated from these two approximately perpendicular views. All parameters were computed as the mean of 3–5 measurements in patients with atrial fibrillation (AF).

**Three-dimensional (3D)-transesophageal echocardiography (TEE)**

The 3D-TEE was performed using an EPIQ7 system with a fully sampled matrix array transducer (X8-2t Live 3D-TEE transducer, Philips), allowing high-volume 3D-imaging. Since TEE employs higher-frequency transducers, which directly enhances spatial resolution^21^, it is considered that TEE is more suitable than TTE for the comprehensive evaluation of tricuspid valve structure.​ The procedure was performed under sedation with diazepam and pentazocine and occasionally with intravenous thiamylal sodium. Based on the recommended depth of anesthesia for endoscopic sedation^22^, moderate sedation or lighter levels of consciousness were aimed for during TEE, ensuring patients remained responsive to verbal commands. Volume datasets were acquired during breath-holding to minimize stitching artifacts, using the multi-beat 3D-zoom mode in the mid-esophageal oblique short-axis view, coronary sinus view, or transgastric RV inflow view, focusing on the tricuspid valve (TV) (median frame rate: 59 Hz; interquartile range [IQR]: 29–100 Hz). In patients with AF, the Live 3D-zoom mode with low-beat volume acquisition was employed to prevent stitching artifacts.

**3D Analysis of the TV**

TV segmentation was performed using a comprehensive multi-step approach. Initially, the precise landmarks of the tricuspid annulus, including the anterior, posterior, septal, and free-wall regions, were carefully selected on a reference frame during mid-systole. This selection uses the four-chamber and two-chamber views to ensure accuracy. Subsequently, TV leaflets were automatically detected using advanced imaging techniques. When the traced annuli and leaflets were inaccurate, manual corrections were made to enhance precision. Upon approval of the final segmentation, TV measurements were automatically calculated, facilitating further analysis.^23^

**Supplementary Figure 1.**


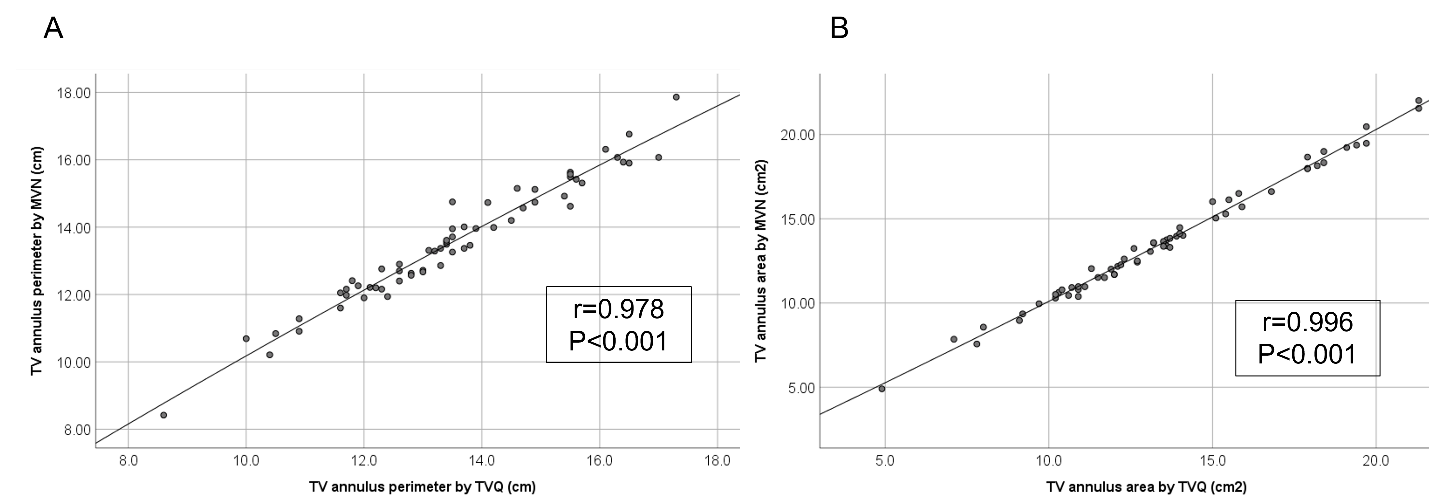


(A) Comparison of the TV annulus perimeter measured using 4D Auto TVQ application and QLAB mitral valve navigator software. (B) Comparison of the TV annulus area measured using 4D Auto TVQ application and QLAB mitral valve navigator software.

Strong correlations were identified between the measurements from the two software programs for the TV annulus perimeter (r = 0.978, p < 0.001) and the TV annulus area (r = 0.996, p < 0.001).
